# Group Search Optimizer for the Mobile Location Management Problem

**DOI:** 10.1155/2014/430705

**Published:** 2014-08-11

**Authors:** Dan Wang, Congcong Xiong, Wei Huang

**Affiliations:** ^1^School of Computer Science and Information Engineering, Tianjin University of Science & Technology, Tianjin 300222, China; ^2^State Key Laboratory of Virtual Reality Technology and Systems, Beihang University, Beijing 100191, China; ^3^School of Computer and Communication Engineering, Tianjin University of Technology, Tianjin 300384, China

## Abstract

We propose a diversity-guided group search optimizer-based approach for solving the location management problem in mobile computing. The location management problem, which is to find the optimal network configurations of management under the mobile computing environment, is considered here as an optimization problem. The proposed diversity-guided group search optimizer algorithm is realized with the aid of diversity operator, which helps alleviate the premature convergence problem of group search optimizer algorithm, a successful optimization algorithm inspired by the animal behavior. To address the location management problem, diversity-guided group search optimizer algorithm is exploited to optimize network configurations of management by minimizing the sum of location update cost and location paging cost. Experimental results illustrate the effectiveness of the proposed approach.

## 1. Introduction

Mobile location management becomes more and more important with the rapid growing mobile network in recent years. Lots of novel applications for data transfer based on fax, voice, and many other mobile services need to be taken into account for making the next generation of mobile network, which supports a basic global personal communication network. To support such applications, mobility management has to be considered when designing infrastructure for wireless mobile networks [1–4].

Reporting cells scheme is conventionally a kind of location management strategies for implementing effective location management [5]. In this case, the minimization of location management cost is considered to decide the reporting cells configuration and finding the optimal set of the reporting cells can be regarded as an optimization problem. However, it is the fact that such an optimization problem has been proven an NP-complete problem [6]. To address this problem, many optimization approaches have been proposed in the past decades. Pioneering work by Taheri and Zomaya [7, 8], Demestichas et al. [9], and Yuen and Wong [10] has studied the mobile location management problem using different evolutionary optimization approaches. In a nutshell, evolutionary algorithms have proven to be very useful for optimizing such problems.

Recently, group search optimizer (GSO) [11] inspired by animal behavior is proposed as a new evolutionary algorithm, and it obtains effective competitive performance when solving some optimization problems [12, 13]. However, such animal behavior-inspired algorithm is essentially stochastic search techniques like many other evolutionary algorithms. Such random mechanism leads to premature convergence problem due to purely random resampling or selections of individuals from a given population, which may lead to revisiting nonproductive regions of the search space [12]. This drawback sometimes limits the application of GSO when dealing with online problems or real-time constraints.

In this paper, we propose a diversity-guided group search optimizer (DGSO) to deal with the location management in mobile computing. In the design of DGSO, diversity guidance is used to prevent the fact that the reducing diversity takes place too early. With the use of diversity guidance, selection operator keeps the diversity of population. Moreover, the proposed DGSO is employed to optimization of the set of the reporting cells in the mobile location management problem.

## 2. Mobile Location Management Problem

In cellular network systems, the mobile location management problem consists of two basic operations, namely, location update and location inquiry. The location update corresponds to the notification of current location, performed by mobile terminals when they change their location in the network, while the location inquiry is the operation of determining the location of the mobile terminal, which is executed by the network when it tries to direct an incoming call to the user [5].

As to mobile location management problem, here we consider minimizing the location management cost, which includes location update operation and location paging operation.

**Figure 1:**
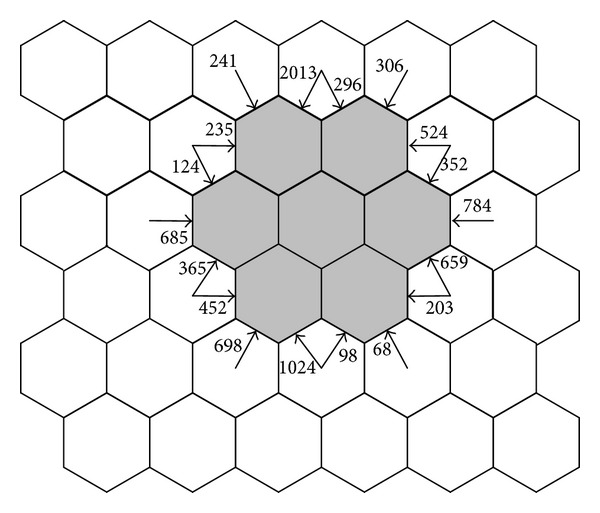
Entering flow of users and call arrival.

The location update cost (LUC) corresponds to the cost of user which performs an update when it moves from the current location area into another location area. If we consider a typical GSM network as shown in Figure 1, it is easy to see the total number of users that enter in the white location area. The location update cost can be calculated as the following expression:

(1) NLUC=2013+296+306+524+352+784+659 +203+68+98+1024+698+452 +365+685+124+235+241=9127.

**Figure 2:**
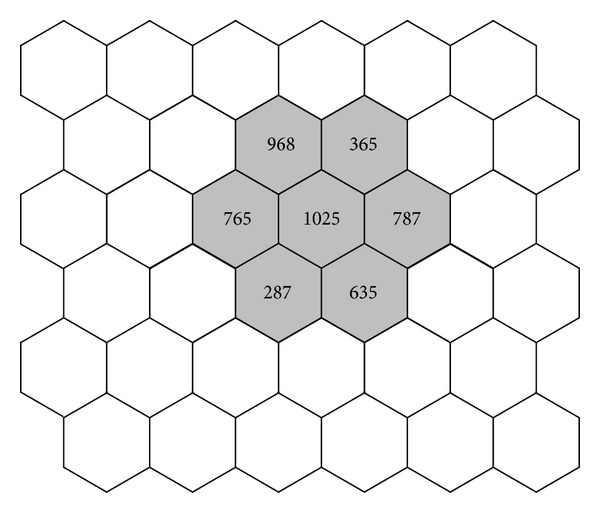
Entering flow of users for an instant locafiguretion area.

The location paging operation cost is caused by the network when it tries to locate a user's mobile terminal, during the location inquiry, and normally the number of paging transactions is directly related to the number of incoming calls [7].  Figure 2 shows an example of incoming calls to the white location area. The paging cost can be obtained as follows:

(2) $$\begin{array}{c} N_{P}=968+365+765+1025+787+287+635=4832. \end{array}$$

Generally, the total cost of location areas is computed by using the following formula [7]:

(3) $$\begin{array}{c} \text{Cost}=\beta \times N_{\text{LUC}}+N_{P}. \end{array}$$

The cost of a location update is normally considered to be 10 times greater than the cost of paging; that is, *β* = 10 [1, 14–16]. With the combination of location update cost (1) and location paging costs (2), the total cost by (3) can be calculated as follows:

(4) $$\begin{array}{c} \text{Cost}=10\times 9127+4832=96102. \end{array}$$

In the reporting cells scheme the location updates only are preformed when a mobile user enters in a reporting cell and the vicinity factor of each cell must be considered [1]. In this case, the genetic formula given by (3) must be readjusted and it is formulated as [15]

(5) $$\begin{array}{c} \text{Cost}=\beta \times \underset{i\in S}{\sum }N_{\text{LUC}}\left(i\right)+\overset{N}{\underset{i=0}{\sum }}N_{P}\left(i\right)\times V\left(i\right), \end{array}$$

where *N* is the number of location updates related to the reporting cell *i*, *S* represents the subset of reporting cells, *N*_*p*_ is the number of incoming calls of cell *i*, *N* is the number of cells that compound the mobile network configuration, and *V* is the vicinity factor attributed for cell *i*.

## 3. Group Search Optimizer Algorithm

Like the swarm intelligence evolutionary algorithms, GSO is also a population-based optimization algorithm. The population is called a* group* and each individual in the population is called a* member *[17, 18].

### 3.1. Main Operators in GSO

The GSO algorithm that is based on the producer-scrounger (PS) model has three basic operators. The main operators in GSO algorithm are as follows.

#### 3.1.1. Initialization

For convenience, the related symbols are summarized as follows [19]: in a *n*-dimension search space, the *i*th member at the *k*th iteration has a current position *X*_*i*_^*k*^ ∈ *R* ^*n*^; a head angle *φ*_*i*_^*k*^ = (*φ*_*i*_1__^*k*^,…, *φ*_*i*_(*n*−1)__^*k*^) ∈ *R* ^*n*−1^, and a search direction of the *i*th member which is a unit vector *D*_*i*_^*k*^(*φ*_*i*_^*k*^) = (*d*_*i*_1__^*k*^,…, *d*_*i*_*n*__^*k*^) ∈ *R* ^*n*^; the unit vector can be calculated as [20]

(6) di1k=∏q=1n−1cos⁡⁡(φiqk)dijk=sin⁡(φi(j−1)k)∏q=jn−1cos⁡⁡(φiqk) (j=2,…,n−1)dink=sin⁡(φi(n−1)k),

where the *X*_*i*_^*k*^, *φ*_*i*_^*k*^, and *d*_*i*_^*k*^ are randomly generated for the initialization of group.

For simplifying the algorithm computational capability, we assume that there is only one producer (here we select the best member as the producer) at each iteration for calculating the group member's fitness value.

#### 3.1.2. Producing

When the only producer is selected, it scans the environment for searching the resource. In the GSO algorithm [18], the scanning field was characterized by the maximum pursuit angle *θ*_max⁡_ and the maximum pursuit distance *l*_max⁡_. The *k*th iteration of the *X*_*p*_ behaves as follows.

First, the producer will randomly scan three points for getting the best point [21]: at zero degree, the point represents as follows:

(7) $$\begin{array}{c} X_{z}=X_{p}^{k}+r_{1}l_{\max}D_{p}^{k}\left(\varphi^{k}\right). \end{array}$$

In the right hand side hypercube, the point is expressed as follows:

(8) $$\begin{array}{c} X_{r}=X_{p}^{k}+r_{1}l_{\max}D_{p}^{k}\left(\varphi^{k}+\frac{r_{2}\theta_{\max}}{2}\right). \end{array}$$

In the left hand hypercube, the point represents as follows:

(9) $$\begin{array}{c} x_{l}=X_{p}^{k}+r_{1}l_{\max}D_{p}^{k}\left(\varphi^{k}-\frac{r_{2}\theta_{\max}}{2}\right), \end{array}$$

where *r*_1_ is the normally distributed random number and *r*_2_ is the distributed random sequence in the range between 0 and 1.

Second, the producer will find the best point by calculating the fitness value. If the producer cannot find the better resource than the current position, the current position is considered the best point; otherwise, the current point will be changed, and the new angle is generated by using the following formula:

(10) $$\begin{array}{c} \varphi^{k+1}=\varphi^{k}+r_{2}\alpha_{\max}, \end{array}$$

where *α*_max⁡_ denotes the maximum turning angle.

Assuming that it could not find the better resource than the current position after the *a*th iterations, we have the angle:

(11) $$\begin{array}{c} \varphi^{k+a}=\varphi^{k}. \end{array}$$

#### 3.1.3. Scrounging

Randomly select 80% from the rest of members to perform scrounging.

#### 3.1.4. Dispersion

In the disperse operator, we use the random walks [22–24] for searching the distributed resources. At the *k*th iteration, it generates a random head angle *φ*_*i*_ using (5); it chooses a random distance

(12) $$\begin{array}{c} l_{i}=a\cdot r_{1}l_{\max} \end{array}$$

and the new point is

(13) $$\begin{array}{c} X_{i}^{k+1}=X_{i}^{k}+l_{i}D_{i}^{k}\left(\varphi^{k+1}\right). \end{array}$$

### 3.2. Diversity-Guided GSO

Like the most of population-based algorithms, the initialization* group* (population) and the searching operators are started with* random guesses* due to the lack of a priori information. To alleviate this problem, diversity guidance is explored here for increasing the diversity of initialization group and the generation jumping embedded in the GSO algorithm. Here the variable “diversity” of group (population) [25] is calculated as follows:

(14) $$\begin{array}{c} \text{diversity}=\frac{1}{\left|S\right|•\left|L\right|}•\overset{\left|S\right|}{\underset{i=1}{\sum }}\sqrt{\overset{N}{\underset{j=1}{\sum }}{\left(p_{ij}-{\overset{-}{p}}_{j}\right)}^{2}}, \end{array}$$

where *S* is the group (population), |*S*| is the size of group, |*L*| is the length of longest the diagonal in the search space, *N* is the dimension of the problem, *p*_*ij*_ is the *j*th value of the *i*th individual, and ${\overset{-}{p}}_{j}$ is the *j*th value of the average point $\overset{-}{p}$; it has also stressed that the diversity measure is independent of group size, the dimensionality of the problem, and the search range in each dimension. For convenience, in this paper *w*_1_, *w*_2_ are set as 0.33 and 0.66, respectively. The procedure of diversity-guided operator is realized as follows.

**Case I.**

If diversity is less than dLow, set *l*_max⁡_ = *w*_1_∗*l*_max⁡_.

**Case II.**

If diversity is greater than dHigh, do nothing (*l*_max⁡_ = *l*_max⁡_).

**Case III.**

If diversity is greater than or equal to dLow, and diversity is less than dHigh, set *l*_max⁡_ = *w*_2_∗*l*_max⁡_.

Here *l*_max⁡_ is defined in formula (2), (3), and (4); dLow, dHigh, *w*_1_, and *w*_2_ are given numbers.

Furthermore, the procedure of DGSO can be outlined as shown in Algorithm 1.

## 4. Experimental Studies

This section reports the experimental results of the proposed DGSO for solving the location management problem in mobile computing. We compared the performance of DGSO with the performance achieved by some “common” algorithms [8] such as genetic algorithm, tabu search, and the ant colony algorithm, respectively. All algorithms are tested based on three test networks with different sizes, and each algorithm is run 200 times for each test network. Furthermore, to compare with the results reported in the literatures, the* cost per call arrival* [26] divided the total cost by the total number of call arrivals is used as the fitness (objective value).

The parameters of DGSO and GSO are as follows [8]: the initialization of population is generated uniformed at random; the initial head angle is set to be *π*/4; the maximum pursuit angle *θ*_max⁡_ is *π*/*a* ^2^, where *a* is given by $\text{Round}(\sqrt{n+1})$ and *n* is the dimension of solutions; the maximum turning angle is *θ*_max⁡_/2.

### 4.1. Test Network with 16 Cells

The first experiment is implemented based on a 4 × 4 size network, whose data set is shown in Table 1 [26]. Here the *w*_*mi*_ and *w*_*ci*_ denote a movement weight and call arrival weight for the cell *i*, respectively.

**Figure 3:**
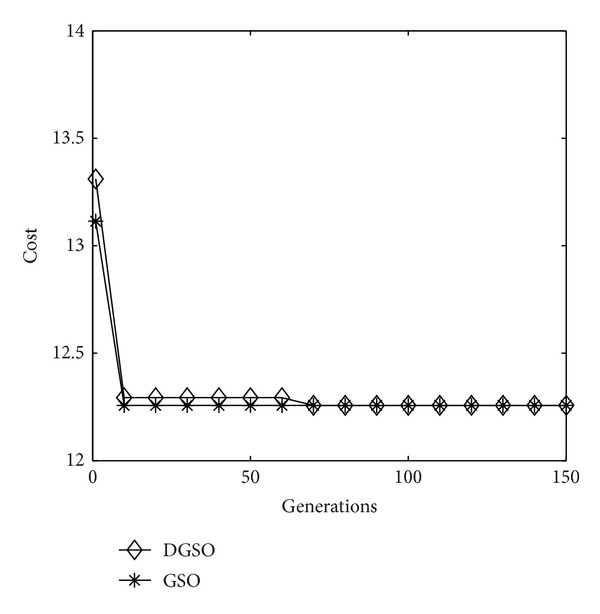
Performance index in successive generations (4 × 4 network).

**Figure 4:**
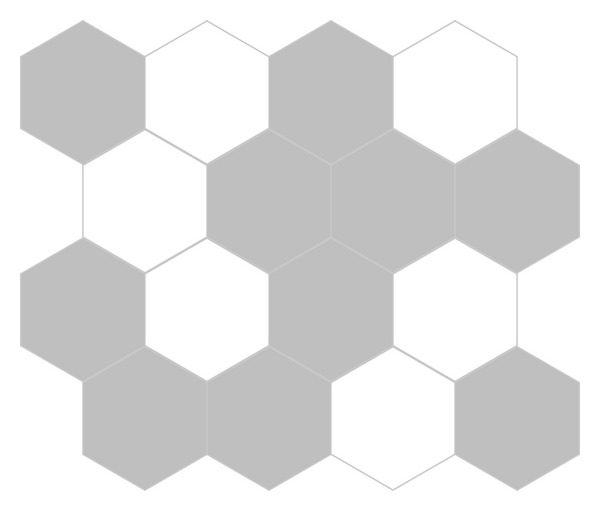
4 × 4 test network optimal solution with reporting cells configuration.

The algorithm presented earlier is adapted here to solve the problem.  Figure 3 shows the process of optimization with the successive generations by using the GSO and DGSO, respectively.  Table 2 summarizes the comparison results of running the algorithms. It is evident that the proposed DGSO and GSO lead to smaller mean value in comparison with the GA; however, the two GSOs obtain the same results as the AC, TDE, and HCDE. That is, the above five algorithms obtain the optimal solution with reporting cells configuration shown in Figure 4.

### 4.2. Test Network with 36 Cells

**Figure 5:**
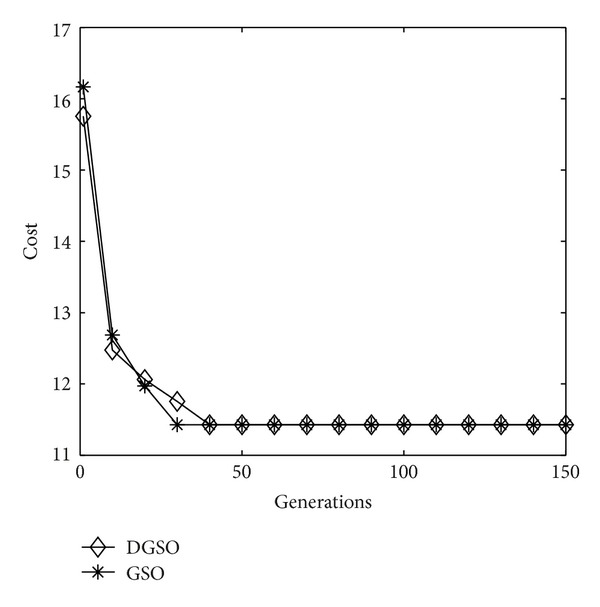
Performance index in successive generations (6 × 6 network).

For the second experiment, a 6 × 6 size network is utilized, and Table 3 provides the data set [26]. After running the GSOs with 150 generations, we get the comparison of GSO and DGSO shown in Figure 5. It shows that both DGSO and GSO have low cost and rapid convergence. The comparison results of some common algorithms are summarized in Table 4.

**Figure 6:**
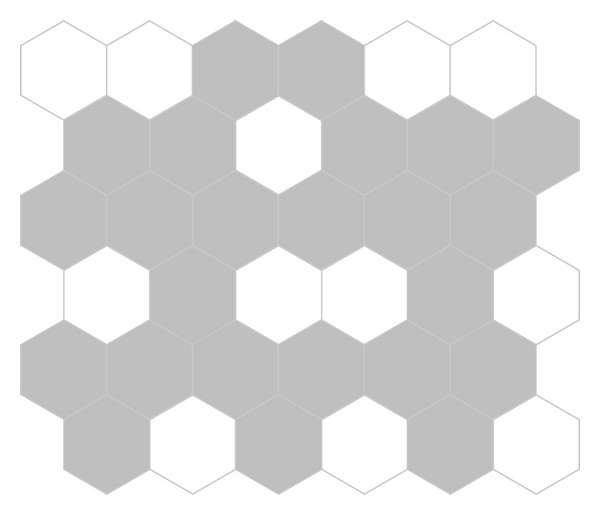
6 × 6 test network optimal solution with reporting cells configuration.

As shown in Table 4, the minimization cost of DGSO is only 11.425, while the best optimal cost reported in previous literatures is 11.471. It appears that the proposed DGSO outperforms several previous algorithms known in the literature.  Figure 6 depicts the optimal solution with reporting cells configuration.

### 4.3. Test-Network with 64 Cells

**Figure 7:**
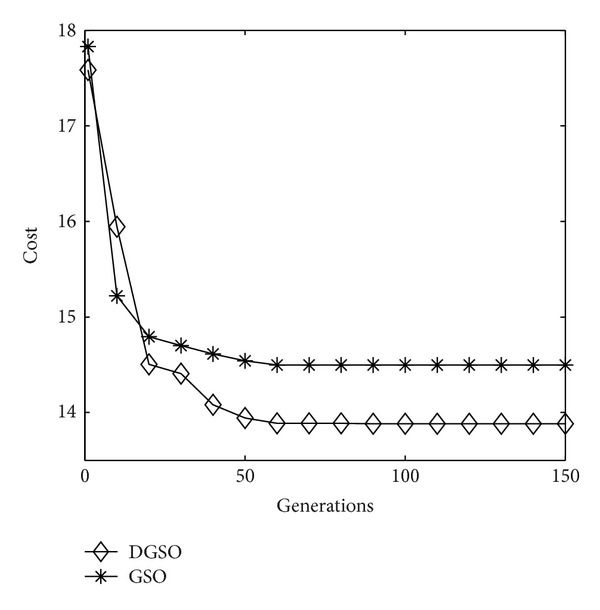
Performance index in successive generations (8 × 8 network).

The last experiment is carried out on a 8 × 8 size test network, whose data set is given in Table 5 [26].  Figure 7 describes the process of optimization using GSO and DGSO with a size of 100 populations and 150 generations, respectively. It shows that DGSO has less cost and rapid convergence in comparison with GSO. In the 4 × 4 and 6 × 6 size test networks, the results of DGSO are the same as the GSO due to the relative small size of test network. With the increase of the size of network, we can safely expect that the DGSO may obtain better performance than the original GSO.

**Figure 8:**
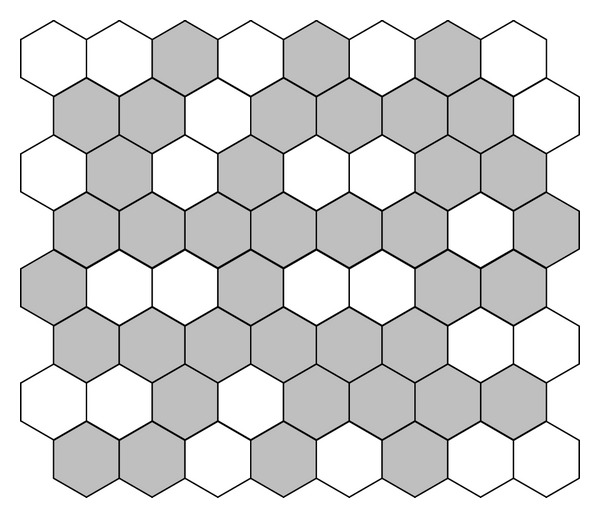
8 × 8 test network optimal solution with reporting cells configuration.

The cost of the proposed DGSO is also compared with the performance of some other algorithms; refer to Table 6. The minimum value of the DGSO and some other algorithms are almost the same; however, it is evident that the performance of the proposed DGSO is better in sense of its maximum value and deviation.  Figure 8 describes the optimal solution with reporting cells configuration.

## 5. Conclusions

This paper presents the DGSO and shows its application to mobile location management problem. Two important aspects are worth highlighting here.

1. We proposed the design of DGSO with the aid of diversity operator. Experimental results based on 8 × 8 test network demonstrate the contribution of diversity-guided operator.
2. We employ GSO and DGSO to deal with the mobile location management problem. Experimental results show that DGSO exhibits good performance in the comparison with some other algorithms reported in the literatures.

## Figures and Tables

**Algorithm 1:**
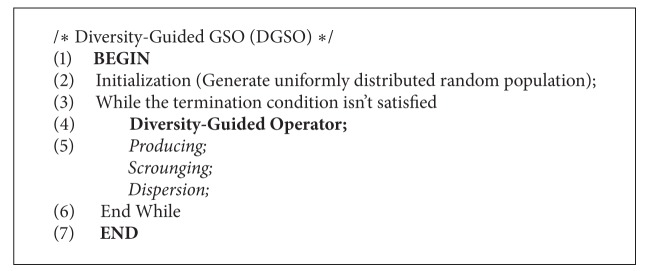
Procedure of diversity-guided GSO (DGSO).

**Table 1:** Cells attribute for the 4 × 4 test network.

Cells	*w*_*mi*_	*w*_*ci*_	Cells	*w*_*mi*_	*w*_*ci*_	Cells	*w*_*mi*_	*w*_*ci*_	Cells	*w*_*mi*_	*w*_*ci*_
1	518	517	5	1617	642	9	445	251	13	307	25
2	774	573	6	472	951	10	2149	224	14	385	540
3	153	155	7	650	526	11	1658	841	15	1346	695
4	1696	307	8	269	509	12	952	600	16	572	225

**Table 2:** Results for the 4 × 4 network.

Algorithms	Average value	Deviation	Minimum value	Deviation	Maximum value	Deviation
GA [26]	12.253	0.006%	12.252	0.000%	12.373	0.986%
TS [26]	12.252	0.000%	12.252	0.000%	12.252	0.000%
AC [26]	12.252	0.000%	12.252	0.000%	12.252	0.000%
TDE [27]	12.252	0.000%	12.252	0.000%	12.252	0.000%
HCDE [27]	12.252	0.000%	12.252	0.000%	12.252	0.000%
Our methods						
GSO	12.252	0.000%	12.252	0.000%	12.252	0.000%
DGSO	12.252	0.000%	12.252	0.000%	12.252	0.000%

**Table 3:** Cells attribute for the 6 × 6 test network.

Cells	*w*_*mi*_	*w*_*ci*_	Cells	*w*_*mi*_	*w*_*ci*_	Cells	*w*_*mi*_	*w*_*ci*_	Cells	*w*_*mi*_	*w*_*ci*_
1	1039	714	10	296	221	19	1945	462	28	1342	370
2	1476	120	11	793	856	20	1368	682	29	814	721
3	262	414	12	317	652	21	1850	241	30	747	769
4	442	639	13	507	238	22	1131	700	31	146	17
5	1052	419	14	603	964	23	236	23	32	904	265
6	1902	332	15	1479	789	24	1622	827	33	359	958
7	444	494	16	756	457	25	16	328	34	1729	191
8	1103	810	17	695	708	26	332	255	35	190	551
9	1829	546	18	356	825	27	1203	393	36	1907	467

**Table 4:** Results for the 6 × 6 network.

Algorithms	Average value	Deviation	Minimum value	Deviation	Maximum value	Deviation
GA [26]	11.511	0.343%	11.471	0.000%	12.030	4.867%
TS [26]	11.471	0.000%	11.471	0.000%	11.471	0.000%
AC [26]	11.472	0.007%	11.471	0.000%	11.573	0.883%
TDE [27]	11.471	0.000%	11.471	0.000%	11.471	0.000%
HCDE [27]	11.471	0.000%	11.471	0.000%	11.471	0.000%
Our methods						
GSO	11.456	0.853%	11.426	0.000%	11.471	0.289%
DGSO	11.432	0.676%	11.426	0.000%	11.471	0.795%

**Table 5:** Cells attribute for the 8 × 8 test network.

Cells	*w*_*mi*_	*w*_*ci*_	Cells	*w*_*mi*_	*w*_*ci*_	Cells	*w*_*mi*_	*w*_*ci*_	Cells	*w*_*mi*_	*w*_*ci*_
1	553	968	17	626	184	33	121	952	49	524	345
2	907	745	18	104	787	34	1410	367	50	1400	135
3	515	827	19	1408	319	35	1011	132	51	393	175
4	1965	705	20	1256	25	36	1298	439	52	1272	596
5	1336	902	21	1637	934	37	1634	134	53	1197	677
6	1318	498	22	1950	414	38	1750	153	54	462	283
7	1292	807	23	101	104	39	1948	612	55	548	139
8	1789	62	24	539	881	40	662	216	56	500	307
9	541	331	25	655	694	41	700	878	57	113	272
10	1071	212	26	131	793	42	765	957	58	47	931
11	1759	787	27	1227	955	43	756	363	59	1676	38
12	1416	664	28	450	126	44	436	820	60	1017	896
13	1413	938	29	470	268	45	672	362	61	1307	164
14	1224	719	30	1081	96	46	822	356	62	499	78
15	484	794	31	1714	285	47	1912	637	63	1451	303
16	543	543	32	308	368	48	1402	626	64	1606	578

**Table 6:** Results for the 8 × 8 network.

Algorithms	Average value	Deviation	Minimum value	Deviation	Maximum value	Deviation
GA [26]	14.005	1.619%	13.782	0.000%	14.617	6.454%
TS [26]	13.791	0.071%	13.782	0.000%	13.999	1.580%
AC [26]	14.107	2.361%	13.801	0.141%	14.407	4.539%
TDE [27]	13.889	0.776%	13.782	0.000%	14.093	2.256%
HCDE [27]	13.788	0.043%	13.782	0.000%	13.892	0.798%
Our methods						
GSO	13.791	0.497%	13.782	0.000%	14.102	2.321%
DGSO	13.780	0.037%	13.782	0.000%	13.883	0.786%
